# Clinicopathologic implications of immune classification by PD-L1 expression and CD8-positive tumor-infiltrating lymphocytes in stage II and III gastric cancer patients

**DOI:** 10.18632/oncotarget.15465

**Published:** 2017-02-17

**Authors:** Jiwon Koh, Chan-Young Ock, Jin Won Kim, Soo Kyung Nam, Yoonjin Kwak, Sumi Yun, Sang-Hoon Ahn, Do Joong Park, Hyung-Ho Kim, Woo Ho Kim, Hye Seung Lee

**Affiliations:** ^1^ Department of Pathology, Seoul National University College of Medicine, Jongno-gu, Seoul 03080, Republic of Korea; ^2^ Department of Internal Medicine, Seoul National University Hospital, Jongno-gu, Seoul 03080, Republic of Korea; ^3^ Department of Internal Medicine, Seoul National University Bundang Hospital, Bundang-gu, Seongnam-si, Gyeonggi-do 13620, Republic of Korea; ^4^ Department of Pathology, Seoul National University Bundang Hospital, Bundang-gu, Seongnam-si, Gyeonggi-do 13620, Republic of Korea; ^5^ Department of Pathology, Soonchunhyang University Seoul Hospital, Yongsan-gu, Seoul 04401, Republic of Korea; ^6^ Department of Surgery, Seoul National University Bundang Hospital, Bundang-gu, Seongnam-si, Gyeonggi-do 13620, Republic of Korea

**Keywords:** gastric cancer, programmed cell death 1 ligand 1, tumor-infiltrating lymphocytes, cancer microenvironment

## Abstract

We co-assessed PD-L1 expression and CD8^+^ tumor-infiltrating lymphocytes in gastric cancer (GC), and categorized into 4 microenvironment immune types. Immunohistochemistry (*PD-L1*, CD8, Foxp3, E-cadherin, and p53), *PD-L1* mRNA *in situ* hybridization (ISH), microsatellite instability (MSI), and EBV ISH were performed in 392 stage II/III GCs treated with curative surgery and fluoropyrimidine-based adjuvant chemotherapy, and two public genome databases were analyzed for validation. PD-L1^+^ was found in 98/392 GCs (25.0%). The proportions of immune types are as follows: PD-L1^+^/CD8^High,^ 22.7%; PD-L1^−^/CD8^Low^, 22.7%; PD-L1^+^/CD8^Low^, 2.3%; PD-L1^−^/CD8^High^, 52.3%. PD-L1^+^/CD8^High^ type accounted for majority of EBV^+^ and MSI-high (MSI-H) GCs (92.0% and 66.7%, respectively), and genome analysis from public datasets demonstrated similar pattern. PD-L1^−^/CD8^High^ showed the best overall survival (OS) and PD-L1^−^/CD8^Low^ the worst (*P* < 0.001). PD-L1 expression alone was not associated with OS, however, PD-L1^−^/CD8^High^ type compared to PD-L1^+^/CD8^High^ was independent favorable prognostic factor of OS by multivariate analysis (*P* = 0.042). Adaptation of recent molecular classification based on EBV, MSI, E-cadherin, and p53 showed no significant survival differences. These findings support the close relationship between PD-L1/CD8 status based immune types and EBV^+^, MSI-H GCs, and their prognostic significance in stage II/III GCs.

## INTRODUCTION

Gastric cancer (GC) is the fifth most common cancer worldwide [1], the third most common cancer in South Korea [2], and one of the leading causes of cancer-related death worldwide [3]. The close relationship between GC carcinogenesis and chronic inflammation caused by *Helicobacter pylori* and Epstein-Barr virus (EBV) infection has been investigated [4, 5], and this unique immune environment is expected to be an effective target of therapy [6].

Clinical trials of immune checkpoint inhibitors have shown favorable outcomes in some solid tumors, including GC [7–9]. Currently, cell surface expression of PD-L1, as assessed by immunohistochemistry (IHC), is a predictive factor for the response to immune checkpoint inhibitors; however, not all patients benefit from this therapy [10]. Therefore, recent studies have focused on how to predict which patients would clinically benefit from cancer immunotherapy and what lies beyond the mechanism of immune escape. A schematic of the tumor microenvironment immune type (TMIT) was developed for better understanding of immune microenvironment. The classification is based on the expression of PD-L1 and tumor-infiltrating lymphocytes (TILs) and consists of four types as follows: type I (PD-L1^+^/TIL^High^, adaptive immune resistance), type II (PD-L1^−^/TIL^Low^, immune ignorance type), type III (PD-L1^+^/TIL^Low^, intrinsic induction of PD-L1 in the absence of TILs), and type IV (PD-L1^−^/TIL^High^, components other than PD-L1 suppressing the action of TILs) [11]. Though this stratification was criticized for being too simplistic [12], a comprehensive analysis of The Cancer Genome Atlas (TCGA) dataset for various solid tumors, which used *CD8A* expression as a surrogate marker for TILs, revealed significant association between TMIT I (*PD-L1^High^*/*CD8A^High^*) and features like high mutational burden and oncogenic viral infection, suggesting the clinical relevance of this classification [13].

Recent studies suggest that the type of TILs, especially CD8-positive (CD8^+^) cytotoxic T cells, is important for the action of immune checkpoint inhibitors [14]. In GC, EBV-positive (EBV^+^) GCs and MSI-high (MSI-H) GCs are frequently accompanied by heavy infiltration of TILs [15, 16], which may be associated with a favorable response to immune checkpoint blockades. However, other GCs are heterogeneous. Recent studies have proposed that additional markers, including epithelial-mesenchymal transition (EMT) features and *TP53* mutations, could be used for further molecular classification [17, 18], although little is known about these categories from a tumor microenvironment-related perspective.

Considering the importance of both PD-L1 expression and CD8^+^ TILs in defining the tumor immune microenvironment [11–13], we co-assessed PD-L1 expression by immunohistochemistry and the density of CD8^+^ TILs in GC tissue samples and applied the scheme for TMIT classification based on PD-L1 expression/CD8 status. The purpose of this study was to (i) determine the association between TMIT and clinicopathologic features, especially prognostic significance, in stage II and III GCs, as well as the molecular subtypes of GCs, specifically EBV and MSI status, (ii) validate the findings by analysis of publicly available genomic datasets, and (iii) suggest potential biomarkers for better patient selection for immune checkpoint inhibitor therapy.

## RESULTS

### Clinicopathologic characteristics and gene expression status

The baseline clinicopathologic characteristics of the study population are shown in Table 1. The median age was 59 years (range, 20–87 years). Of the 392 patients, 210 (53.6%) were AJCC 7th TNM stage II, and 182 (46.4%) were stage III. Fluoropyrimidine (FP)-based regimen was applied as adjuvant chemotherapy; 336 patients (85.7%) were treated with FP only, and 56 patients (14.3%) were treated with FP and cisplatin. The number of CD8^+^ TILs ranged from 6.90 cells/mm^2^ to 1374.94 cells/mm^2^ with the median value of 195.23 cells/mm^2^. The number of Foxp3^+^ TILs ranged from 1.22 cells/mm^2^ to 785.88 cells/mm^2^ with the median value of 60.12 cells/mm^2^.

**Table 1:** Clinicopathologic characteristics of study population

Tumor microenvironment immune type						
	IPD-L1^+^/CD8^High^	IIPD-L1^−^/CD8^Low^	IIIPD-L1^+^/CD8^Low^	IVPD-L1^−^/CD8^High^	Total	*P*
Age	60 (31–82)	57 (30–87)	68 (43–77)	59 (20–85)	59 (20–87)	0.159
Sex						0.021
Male	70 (27.7%)	51 (20.2%)	9 (3.6%)	123 (48.6%)	253 (64.5%)	
Female	19 (13.7%)	38 (27.3%)	0 (0.0%)	82 (59.0%)	139 (35.5%)	
Lauren classifcation						0.765
Intestinal	37 (25.3%)	29 (19.9%)	7 (4.8%)	73 (50.0%)	146 (37.2%)	
Diffuse	38 (17.8%)	58 (27.1%)	1 (0.5%)	117 (54.7%)	214 (54.6%)	
Mixed	13 (43.3%)	2 (6.7%)	0 (0.0%)	15 (50.0%)	30 (7.7%)	
Indeterminate	1 (50.0%)	0 (0.0%)	1 (50.0%)	0 (0.0%)	2 (0.5%)	
Lymphatic invasion						0.698
Absent	23 (19.7%)	31 (26.5%)	0 (0.0%)	142 (53.8%)	117 (29.8%)	
Present	66 (24.0%)	58 (21.1%)	9 (3.3%)	174 (51.6%)	275 (70.2%)	
Vascular invasion						0.855
Absent	77 (23.5%)	70 (21.4%)	6 (1.8%)	174 (53.2%)	327 (83.4%)	
Present	12 (18.5%)	19 (29.2%)	3 (4.6%)	31 (47.7%)	65 (16.6%)	
Perineural invasion						0.266
Absent	40 (30.5%)	21 (16.0%)	3 (2.3%)	67 (51.1%)	131 (33.4%)	
Present	49 (18.8%)	68 (26.1%)	6 (2.3%)	138 (52.9%)	261 (66.6%)	
pT stage						0.004
pT1	1 (3.6%)	3 (10.7%)	1 (3.6%)	23 (82.1%)	28 (7.1%)	
pT2	22 (28.9%)	7 (9.2%)	1 (1.3%)	46 (60.5%)	76 (19.4%)	
pT3	47 (26.7%)	39 (22.2%)	3 (1.7%)	87 (49.4%)	176 (44.9%)	
pT4	19 (17.0%)	40 (35.7%)	4 (3.6%)	49 (43.8%)	112 (28.6%)	
pN stage						0.839
pN0	10 (21.3%)	11 (23.4%)	1 (2.1%)	25 (53.2%)	47 (12.0%)	
pN1	47 (29.4%)	26 (16.3%)	4 (2.5%)	83 (51.9%)	160 (40.8%)	
pN2	14 (13.1%)	28 (26.2%)	2 (1.9%)	63 (58.9%)	107 (27.3%)	
pN3	18 (23.1%)	24 (30.8%)	2 (2.6%)	34 (43.6%)	78 (19.9%)	
TNM stage						0.110
II	51 (24.3%)	33 (15.7%)	4 (1.9%)	122 (58.1%)	210 (53.6%)	
III	38 (20.9%)	56 (30.8%)	5 (2.7%)	83 (45.6%)	182 (46.4%)	
Chemotherapy regimen						0.177
FP only	80 (23.9%)	64 (19.1%)	9 (2.7%)	182 (54.3%)	335 (85.7%)	
FP + cisplatin	9 (16.1%)	25 (44.6%)	0 (0.0%)	22 (39.3%)	56 (14.3%)	
Foxp3 IHC						< 0.001
High	79 (88.8%)	11 (12.4%)	5 (55.6%)	101 (49.3%)	196 (50.0%)	
Low	10 (11.2%)	78 (87.6%)	4 (44.4%)	104 (50.7%)	196 (50.0%)	
E-cadherin IHC						0.131
N/C	6 (6.7%)	18 (20.2%)	0 (0.0%)	37 (18.0%)	61 (15.6%)	
M	83 (93.3%)	71 (79.8%)	9 (100.0%)	168 (82.0%)	331 (84.4%)	
p53 IHC						0.076
Negative	69 (77.5%)	68 (76.4%)	6 (66.7%)	141 (68.8%)	284 (72.4%)	
Positive	20 (22.5%)	21 (23.6%)	3 (33.3%)	64 (31.2%)	108 (27.6%)	
Total	89 (22.7%)	89 (22.7%)	9 (2.3%)	205 (52.3%)	392 (100.0%)	

Abbreviations: FP, fluoropyrimidine; IHC, immunohistochemistry; N / C, altered expression (negative or cytoplasmic); M, membranous staining; P, *p*-value.

PD-L1 IHC was positive in 98 samples (25.0%), and *PD-L1* mRNA overtranscription (a *PD-L1* mRNA ISH score of 4+) was detected in 14 samples (3.6%). When PD-L1 IHC and mRNA ISH were compared, all cases with mRNA ISH score of 4+ were PD-L1 IHC positive, and the correlation coefficient between the 2 tests was 0.467, which was statistically significant at the 0.01 level (Supplementary Table 1). Altered expression of E-cadherin was detected in 61 of 392 samples (15.6%), and overexpression of p53 was detected in 108 of 392 samples (27.6%).

### Immune and molecular classification of GCs

**Figure 1:**
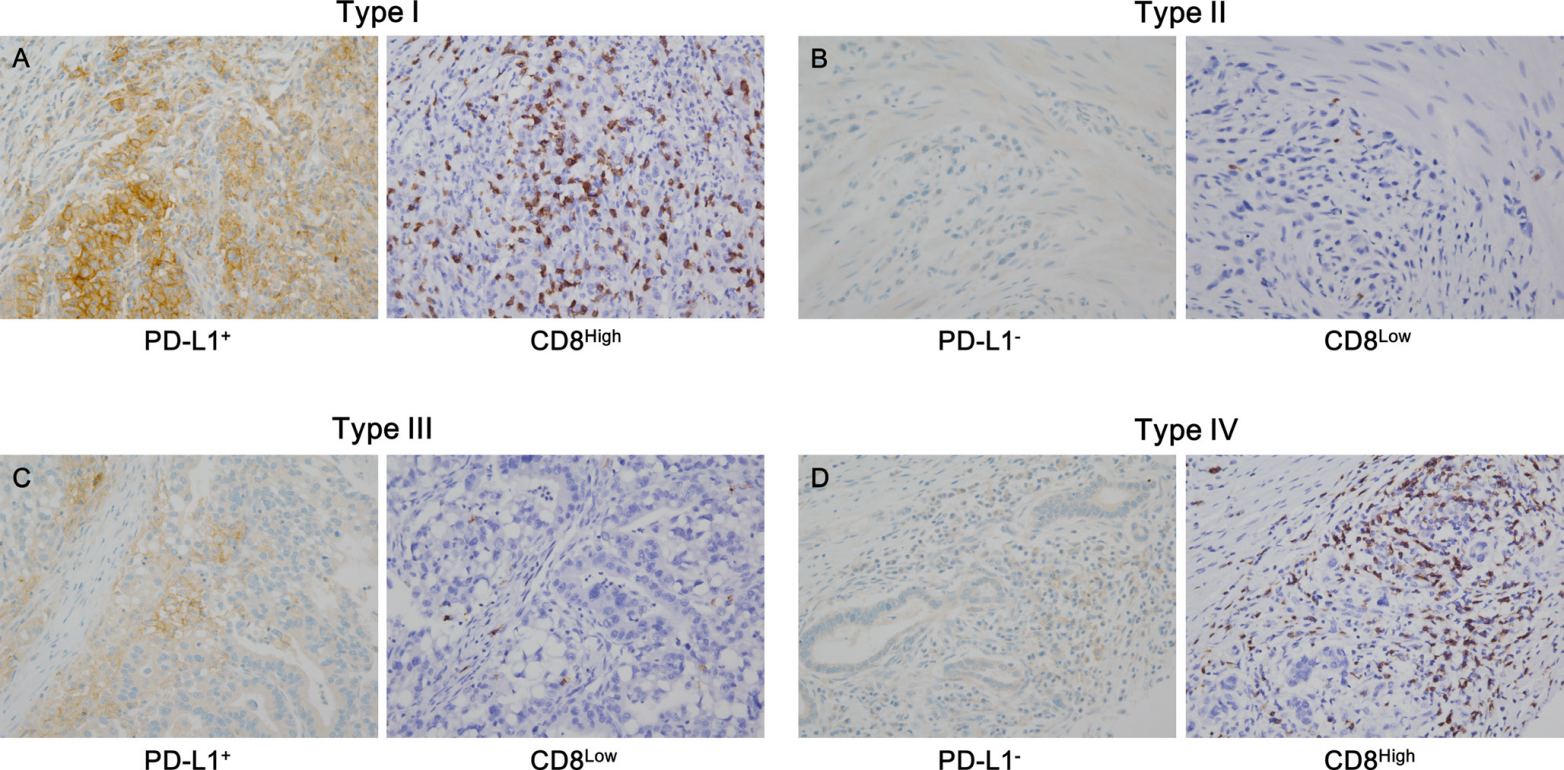
Representative cases in each tumor microenvironment immune type (TMIT) The TMIT classification is as follows: (**A**) type I (PD-L1^+^/CD8^High^), (**B**) type II (PD-L1^−^/CD8^Low^), (**C**) type III (PD-L1^+^/CD8^Low^), and (**D**) type IV (PD-L1^−^/CD8^High^). PD-L1^+^ was defined as PD-L1 membrane staining in more than 5% of tumor cells (A, left; C, left), and CD8^High^ was defined as a density of CD8^+^ tumor infiltrating lymphocytes (TILs) exceeding the 25th percentile (A, right; D, right).

We categorized the study population into TMITs I–IV based on the results of PD-L1 IHC and CD8^+^ TIL density (Figure 1). The number and proportion of each type were as follows: type I (PD-L1^+^/CD8^High^), 89 (22.7%); type II (PD-L1^−^/CD8^Low^), 89 (22.7%); type III (PD-L1^+^/CD8^Low^), 9 (2.3%); and type IV (PD-L1^−^/CD8^High^), 205 (52.3%). Type I showed more male predominance than the other types (*P* = 0.021), and AJCC 7th pT stage was significantly associated with TMIT (*P* = 0.004). In addition, type I was associated with Foxp3^High^ status, and type II was associated with Foxp3^Low^ status (*P* < 0.001). No other significant associations between other features and TMIT classification were observed.

Next, we modified and adapted previously described molecular classification models for GC [17, 18] in our study population, according to the process described in Supplementary Figure 1. The GC cohort was classified into 5 molecular groups: EBV^+^ (group 1), MSI-H (group 2), microsatellite stable (MSS)/MSI-low (MSI-L)/EMT-like (group 3), MSS/MSI-L/p53-IHC^+^ (group 4), and MSS/MSI-L/p53-IHC^−^ (group 5). Of the 392 patients, 25 were in group 1 (6.4%), and 36 were group 2 (9.2%); none of the EBV^+^ GCs showed an MSI-H phenotype, and vice versa. The number of patients in groups 3, 4, and 5 were 105 (26.8%), 73 (18.6%), and 153 (39.0%), respectively.

### Survival analysis

**Figure 2:**
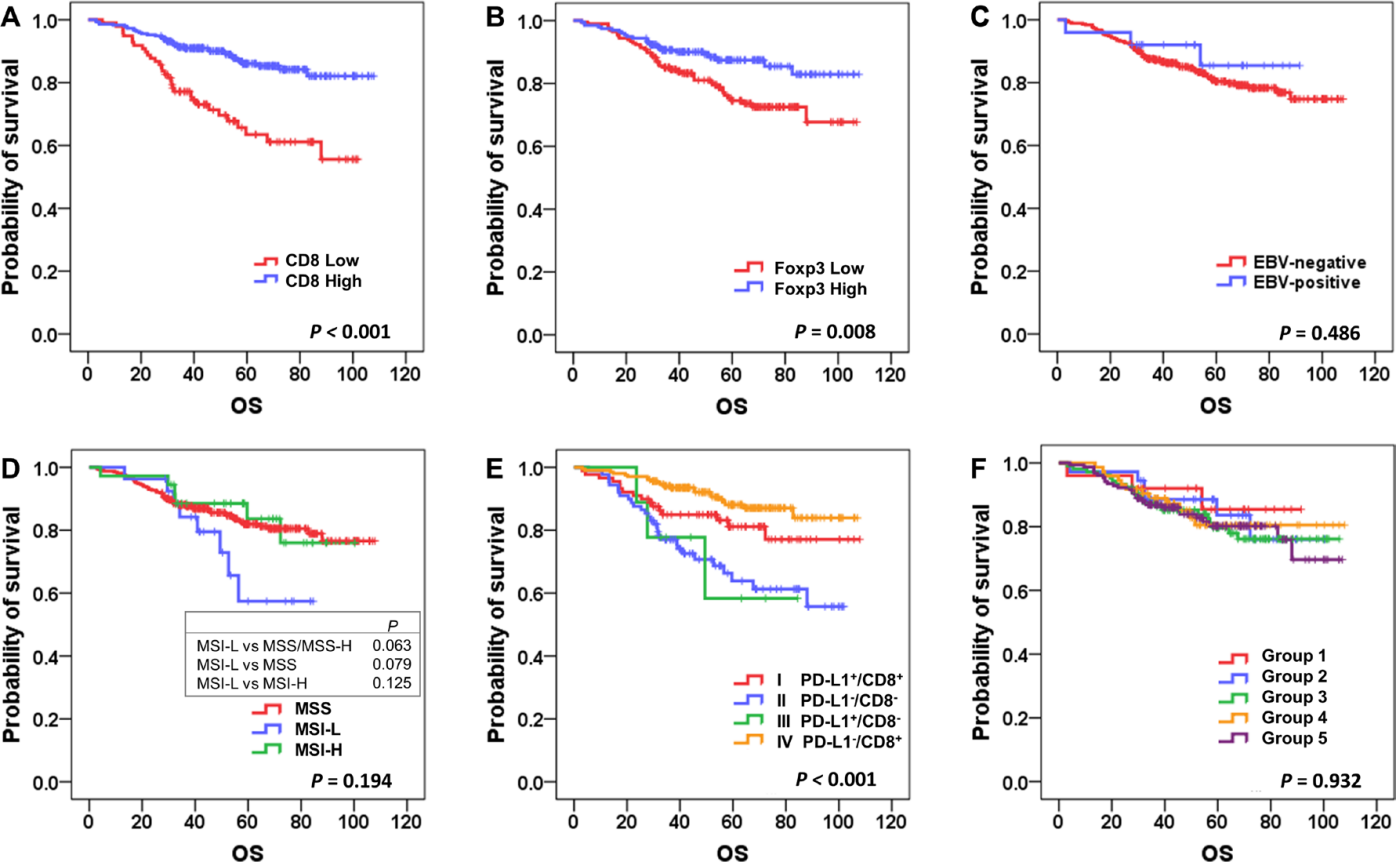
Kaplan-Meier survival analysis of overall survival according to major clinicopathologic features Higher densities of CD8+ and Foxp3+ cells were associated with better overall survival (**A** and **B)**; *P* < 0.001 and *P* = 0.008, respectively), whereas Epstein-Barr virus (EBV) status was not a significant prognostic factor (**C**). Microsatellite instability-low (MSI-L) cases showed poor prognosis compared to others (**D**). There were significant survival differences among the 4 tumor microenvironment immune types (TMITs; **E**; *P* < 0.001), whereas there were no discernible differences according to molecular classification (**F**).

Kaplan-Meier survival analyses were performed, and the results showed that patients in the CD8^High^ group had significantly better overall survival (OS) than the CD8^Low^ group (*P* < 0.001; Figure 2A), and that Foxp3^High^ was associated with better OS (*P* = 0.008; Figure 2B) in stage II and III GC patients with standard treatment. There was no significant survival difference between EBV^+^ and EBV^−^ GCs (*P* = 0.486; Figure 2C). Analysis according to MSI status showed that MSI-L patients had worse OS when compared to MSI-H and MSS patients, with borderline statistical significance (*P* = 0.063; Figure 2D). PD-L1 IHC positivity itself was not significantly associated with survival (*P* = 0.579; Supplementary Figure 2A), and there were no OS differences according to E-cadherin and p53 IHC (*P* = 0.838 and 0.216, respectively; Supplementary Figure 2B and 2C).

We also performed Kaplan-Meier survival analysis according to TMIT and molecular classification. Of the 4 TMITs, type IV (PD-L1^−^/CD8^High^) had the best OS, and type II (PD-L1^−^/CD8^Low^) had the worst OS (*P* < 0.001; Figure 2E). Interestingly, when TMITs I and IV (the CD8^High^ groups) were compared, type IV (PD-L1^−^/CD8^High^) had better OS, with marginal statistical significance (*P* = 0.070). However, according to the molecular classification, no significant survival differences were detected among the 5 groups (*P* = 0.791; Figure 2F).

In addition, we performed survival analysis of disease free survival (DFS) (Supplementary Figure 3), which showed similar results when compared to the analysis of OS; high CD8^+^, Foxp3^+^ cells were associated with better DFS (*P* < 0.001 and *P* = 0.021, respectively), and TMIT IV showed the best DFS while TMIT II was associated with the worst DFS (*P* < 0.001).

Univariate analysis of OS by Cox proportional hazard model showed that age, vascular invasion, perineural invasion, chemotherapy regimen, TNM stage, CD8^+^ TILs, Foxp3^+^ TILs, and TMIT IV are the key clinicopathologic features that are significantly associated with OS (Table 2). By multivariate analysis, older age, the presence of vascular invasion, addition of cisplatin to FP-based chemotherapy, higher TNM stage, and CD8^High^ status were significantly correlated with OS. Furthermore, when compared to the type I and II/III groups, TMIT IV was an independent prognostic factor for OS, with statistical significance (hazard ratios, 2.11 and 2.53; 95% confidence intervals, 1.03–4.33 and 1.42–4.51; and *P* = 0.042 and 0.002, respectively; Table 2, right column).

**Table 2:** Univariate and multivariate analysis of overall survival by Cox proportional hazards model

Variable		Univariate			Multivariate (TMIT)			Multivariate (CD8^+^ TILs)		
		HR	95% CI	*P*	HR	95% CI	*P*	HR	95% CI	*P*
Age		1.03	1.01–1.05	0.002	1.04	1.01–1.06	0.002	1.04	1.01–1.06	0.002
Sex	Female vs male	1.03	0.63–1.67	0.920						
Lymphatic invasion	present vs absent	1.72	0.98–3.09	0.070						
Vascular invasion	present vs absent	3.70	2.29–6.00	< 0.001	1.99	1.19–3.32	0.008	1.97	1.19–3.29	0.009
Perineural invasion	Present vs absent	3.08	1.58–6.01	0.001	1.69	0.83–3.45	0.149	1.63	0.80–3.31	0.179
Chemotherapy regimen	FP only vs FP+C	4.69	2.81–7.82	< 0.001	3.55	2.01–6.26	< 0.001	3.59	2.04–6.33	< 0.001
TNM stage	III vs II	6.12	3.34–11.20	< 0.001	3.10	1.61–5.96	0.001	3.15	1.64–6.08	0.001
PD-L1 IHC	P vs N	1.16	0.69–1.97	0.579						
CD8^+^ TILs	High vs Low	0.34	0.21–0.55	< 0.001				0.46	0.27–0.78	0.004
Foxp3^+^ TILs	High vs Low	0.52	0.32–0.85	0.009	0.87	0.45–1.66	0.668	1.11	0.63–1.98	0.717
EBV status	P vs N	0.67	0.21–2.11	0.489						
MSI status	MSI-L vs MSS	1.92	0.92–4.03	0.085						
	MSI-H vs MSS	0.90	0.39–2.09	0.808						
E-cadherin IHC	M vs N/C	1.07	0.56–2.04	0.838						
p53 IHC	P vs N	1.37	0.83–2.26	0.218						
TMIT	I vs IV	1.80	0.95– 3.44	0.073	2.11	1.03–4.33	0.042			
	II/III vs IV	3.62	2.10–6.24	< 0.001	2.53	1.42–4.51	0.002			
Molecular	group 2 vs 1	1.27	0.32–5.07	0.737						
classification	group 3 vs 1	1.57	0.47–5.27	0.464						
	group 4 vs 1	1.39	0.39–4.91	0.613						
	group 5 vs 1	1.58	0.48–5.19	0.454						

Abbreviations: HR, hazard ratio; CI, confidence interval; FP, fluoropyrimidine; C, cisplatin IHC, immunohistochemistry; P, positive; N, negative; TIL, tumor infiltrating lymphocytes; MSI, microsatellite instability; MSI-L, MSI-low; MSI-H, MSIhigh; MSS, microsatellite stable; M, membranous statining; N/C, altered expression (negative or cytoplasmic); TMIT, tumor microenvironment immune types; P, p-value.

### Relationship between molecular classification and TMIT

To determine the implications of the molecular classification from an immune microenvironment perspective, we compared TMIT and molecular classification. The relationship between the 2 classifications is shown in Table 3. Twenty-three of the 25 (92%) EBV^+^ GCs (group 1) were type I (PD-L1^+^/CD8^High^); none of the EBV^+^ GCs were CD8^Low^, and only 2 (8.0%) EBV^+^ GCs were PD-L1^−^. Similarly, MSI-H GCs (group 2) also had a distinct relationship with TMIT I; 26 of 36 (72.3%) MSI-H cases were PD-L1^+^, and 24 cases (66.7%) were classified as TMIT I. Within group 3, only 4 of 105 (3.8%) cases were TMIT I, and the proportion of TMIT II cases was relatively high (35/105; 33.3%). In groups 4 and 5, the proportion of each TMIT was similar to that from the whole study population.

**Table 3:** Comparison between molecular classification of gastric cancer and tumor microenvironment immune type

Tumor microenvironment immune type						
	IPD-L1^+^/CD8^High^	IIPD-L1^−^/CD8^Low^	IIIPD-L1^+^/CD8^Low^	IVPD-L1^−^/CD8^High^	Total	*P*
**Molecular classification**						< 0.001
Group 1	23	0	0	2	25	
EBV^+^	(92.0%)	(0.0%)	(0.0%)	(8.0%)	(6.4%)	
Group 2	24	5	2	5	36	
MSI-H	(66.7%)	(13.9%)	(5.6%)	(13.9%)	(9.2%)	
Group 3	4	35	0	66	105	
MSS/MSI-L/EMT-like	(3.8%)	(33.3%)	(0.0%)	(62.9%)	(26.8%)	
Group 4	13	13	3	44	73	
MSS/MSI-L/p53-IHC+	(17.8%)	(17.8%)	(4.1%)	(60.3%)	(18.6%)	
Group 5	25	36	4	88	153	
MSS/MSI-L/p53-IHC-	(16.3%)	(23.5%)	(2.6%)	(57.5%)	(39.0%)	
Total	89	89	9	205	392	
	(22.7%)	(22.7%)	(2.3%)	(52.3%)	(100.0%)	

Abbreviations: EBV, Ebstein-Barr virus; MSI-H, microsatellite instability high; MSI-L, microsatellite instability low; MSS, microsatellite stable; EMT, epithelial mesenchymal transition; IHC, immunohistochemistry; P, *p*-value.

### Validation using genomic data from TCGA and SMC cohort

**Figure 3:**
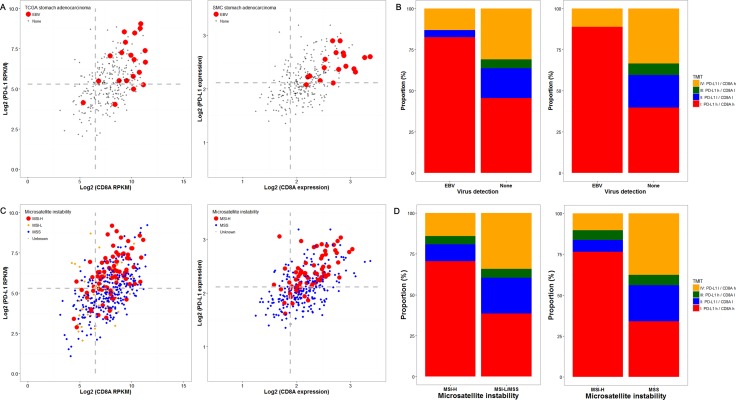
Genomic analysis of the cancer genome atlas (TCGA) and samsung medical center (SMC) cohort datasets according to Epstein-Barr virus (EBV) and microsatellite instability (MSI) status The *PD-L1/CD8A* expression patterns according to EBV status in TCGA (left) and SMC (right) datasets are shown (**A**), and EBV-positivity (red dots) was associated with higher expression of both PD-L1 and *CD8A*. Concordantly, more than 75% of the cases in both datasets were tumor microenvironment immune type (TMIT) I (red box) (**B**). Similarly, MSI-H cases (red dots) were associated with higher *PD-L1/CD8A* expression (**C**) TCGA (left) and SMC (right)), and were thus TMIT I (red box) (**D**).

To validate the aforementioned association between TMIT I and EBV^+^ or MSI-H GCs, we performed analysis of the genomic dataset from TCGA and SMC cohort. As shown in Figure 3A and 3B, the majority of EBV^+^ stomach adenocarcinomas in both datasets were classified as TMIT I (81.1% in TCGA and 88.9% in SMC). Genomic analysis according to MSI status showed that, in accordance with the findings from our tissue samples, most of the MSI-H cases were TMIT I (70.5% in TCGA and 76.5% in SMC), followed by type IV, II, and III (Figure 3C and 3D). In the MSS/MSI-L group, the proportions of each TMIT subtype in TCGA and SMC were similar to the proportions in our 392 patients.

Next, we assessed the expression of *CDH1* in each TMIT to test for an association with molecular classification group 3, which shows EMT-like features. In TCGA dataset, TMIT IV showed the lowest *CDH1* expression, and only the difference between type IV and II showed statistical significance (*P* = 0.017; Supplementary Figure 4A). In contrast, analysis of the SMC dataset showed that *CDH1* expression levels did not differ among the 4 TMITs. We also compared *Foxp3* expression levels in TCGA dataset; type I showed significantly higher expression than the other types (*P* < 0.001; Supplementary Figure 4B), and type IV showed the second highest expression. However, analysis of the SMC cohort did not show any differential *Foxp3* expression among the 4 TMITs.

## DISCUSSION

In this study, we classified a large cohort of stage II and III GC patients who were managed with standard treatment into one of four TMITs, using immunohistochemical assessment of PD-L1 expression and CD8^+^ TIL infiltration as the surrogate markers of the tumor microenvironment (TME). We found that TMIT I (PD-L1^+^/CD8^High^) is closely correlated with EBV infection and MSI-H phenotype than TMIT IV (PD-L1^−^/CD8^High^). Additionally, to validate our results, we analysed datasets from TCGA [19] and the SMC cohort, the latter of which is a mostly Asian population [17]. The results also showed that the EBV^+^ and MSI-H cases in the both datasets were likely to be type I (*PD-L1*^High^/*CD8A*^High^).

Numerous studies have shown that PD-L1 expression is increased in both EBV^+^ and MSI-H GCs [20–22]. Likewise, it is well known that EBV^+^ GCs and MSI-H GCs are associated with heavy lymphocytic infiltration [23, 24]. However, classification of the TME by co-assessment of PD-L1 and TILs had not yet been reported, and a study of a small Western population showed that CD8^+^ T cell-infiltrated GCs are associated with PD-L1 expression [25]. Here, we demonstrated, for the first time, the close association of TMIT I (PD-L1^+^/CD8^High^) with EBV^+^ and MSI-H, compared to type IV (PD-L1^−^/CD8^High^), using both tissue samples and genomic analysis. TMIT I status (PD-L1^+^/CD8^High^) implies the adaptive immune escape responses, and based on many previous studies, there is a good chance that GCs with this signature can be reversed by immune checkpoint blockade [11, 25]. Therefore, we suggest that the type I (PD-L1^+^/CD8^High^) TMIT could serve as a biomarker for a good response to immune checkpoint inhibitors, and that PD-L1 and CD8 TIL status should be evaluated in patients with EBV^+^ or MSI-H GC.

In addition, we also found that the TMIT has prognostic value. TMIT II, which implies the immune ignorant state of tumor microenvironment, shows worse survival outcome compared to highly inflamed status (types I and IV), and this finding is consistent with previous studies from diverse tumor types including GC [21, 23]. Even more important finding from our survival analysis is that OS within the CD8^High^ group differs according to the differential expression of PD-L1; type I (PD-L1^+^/CD8^High^) showed significantly poorer OS than type IV (PD-L1^−^/CD8^High^) by multivariate analysis. From this we could infer that although heavy immune cell infiltration might play the favorable anti-tumor effect in gastric cancer, effective immune evading occurs by expression of PD-L1, possibly resulting in decreased OS. Since PD-L1 expression alone failed to discriminate survival in the total study population, the significant survival difference elucidated by differential PD-L1 expression in the CD8^High^ group strongly suggests that the clinical implication of PD-L1 expression could become more meaningful when interpreted in combination with other components of the TME. Therefore, we suggest co-assessment of both PD-L1 and CD8^+^ TILs as a useful way of defining the TME, which also has a significant prognostic role in stage II and III GC.

Previous studies on the prognostic role of PD-L1 expression in GC showed conflicting results. For example, the most recent study of a large Caucasian cohort of GC showed that PD-L1 expression in tumor and stromal immune cells was associated with better tumor-specific and overall survival [26], while previous studies of an Asian population showed the poor prognostic role of PD-L1 expression [27, 28]. Some authors attributed these discrepant results to differences in the gene signatures between the Asian and Caucasian populations [26, 29]. Apart from ethnicity, we suggest other explanations for the conflicting results. Previous survival analyses of GC according to PD-L1 expression were not performed within the context of the immune microenvironment, as discussed earlier. Furthermore, most studies were performed on heterogeneous populations; that is, patients with cancers of various stages with different clinical settings and treatment strategies. In contrast, our study population was relatively homogenous. In Korea, the 5-year survival rate of the localized gastric cancer patients exceeds 92% [30], therefore, when performing prognostic analysis within the localized gastric cancer group, the chance that the survival outcome of this group may not be directly related to disease itself must be taken into account. In cases of metastatic gastric cancer, the therapeutic approach including chemotherapy regimen widely varies [31], and this heterogeneity may result in possible confounder of the survival analysis. For these reasons, we have restricted the study population into patients with stage II and III GC who were treated by curative surgical resection followed by FP-based adjuvant chemotherapy, with the expectation of causing less bias in the survival analysis. Therefore, we suggest that the prognostic difference found in the present study of stage II and III GCs is notable and very reliable.

Next, we assessed the characteristics that distinguished type IV (PD-L1^−^/CD8^High^) from type I (PD-L1^+^/CD8^High^) GCs. The characteristic immune microenvironment of type IV is activation of the immune-suppressing mechanism other than PD-L1. Therefore, we assessed the role of Tregs in type IV GCs by measuring Foxp3 expression by IHC. Contrary to our assumption, the proportions of Foxp3^High^ and Foxp3^Low^ within the type IV group were approximately equal, and analysis of TCGA and SMC datasets did not show discernible high expression of *Foxp3* in type IV, suggesting that the immune tolerance mechanism in type IV cannot be solely explained by Tregs. Further studies on the various components of the TME in the type IV group, such as tumor-associated macrophages or myeloid-derived suppressor cells should provide a deeper understanding of this topic.

Since the introduction of molecular subtypes of GC in TCGA study, EBV^+^ GCs and MSI-H GCs have been consistently regarded as distinct subtypes [19]. Yet, debates regarding the proper classification of the remaining GCs continue, and little is known about these GCs from an immuno-oncologic perspective. Recently, Setia *et al*. suggested a practical molecular classification model mainly based on IHC analysis of E-cadherin and p53 [18], which we adapted in this study. Based on the previous findings for other types of solid tumors, group 3 (MSS/MSI-L/EMT-like) was expected to be positively associated with PD-L1 expression [32, 33]. However, only 3.1% of group 3 cases (4/105) were PD-L1^+^. This may be due to differences in the biology of GC compared to that of the other cancers for which strong associations were observed. A more precise definition of ‘EMT-like’ is warranted to specify the immuno-oncologic characteristics of this category. When groups 4 (MSS/non-EMT-like/p53-IHC^+^) and 5 (MSS/non-EMT-like/ p53-IHC^−^) were compared according to classification, the proportions of the 4 TMITs within these 2 groups were similar to the proportions in the total population; thus, no distinct immuno-oncologic features according to p53 overexpression were observed.

This study has the limitation of being a retrospective study at a single institution. However, compared to other studies, our study population is a large, relatively homogeneous cohort with restricted confounding factors. The cut-off value for PD-L1^+^ is still a matter of debate; applying different cut-off level for PD-L1 IHC results would inevitably result in different proportions among the TMIT subtypes. However, since there is no general consensus in this topic till nowadays, we have done thorough review of previous studies in pursuit of identifying an ideal cut-off criteria for PD-L1 IHC, and chose our criteria referenced from the most recent studies of GC [20, 25]. In addition, this study was based on the immunostainings on TMA blocks, which enabled us to assess PD-L1 expression in a large cohort of 392 patients. Despite, it is reported that spatial heterogeneity of PD-L1 IHC exists in various types of tumor including non-small cell lung cancer and malignant melanoma [34]. Therefore, even though we have applied 5% positivity as the cut-off for PD-L1 IHC, the possibility of false-negativity should be considered. We have found that to date, there is no consensus on the assessment methods for TILs and their cut-offs. Here, we adapted previously described image-analyzer method [35] to ensure objectivity. For the molecular classification, we could not clearly define the distinct immunologic characteristics of GCs other than EBV^+^ GC and MSI-H GC. This can be attributable to the limitation of IHC itself; we have interpreted p53 IHC into either positive or negative, however, this may not reflect the actual *TP53* gene mutation status accurately. Thus, more precise techniques for assessing genetic mutation and gene expression levels should be warranted in future studies.

In summary, this is the first study to classify a large homogeneous cohort of stage II and III GCs into 4 immune types by co-assessment of 2 key components of TME, PD-L1 expression and CD8^+^ T cell infiltration. We found that EBV^+^ and MSI-H GCs are distinct subtypes that are tightly associated with TMIT I (PD-L1^+^/CD8^High^), and OS within the CD8^High^ group differs according to PD-L1 expression. Therefore, we conclude that co-assessment of PD-L1 and CD8^+^ TILs is clinically relevant, has a possible prognostic role, and warrants further investigation as a predictive marker for immune checkpoint blockade.

## MATERIALS AND METHODS

### Patients and samples

A total of consecutive 406 patients with stage II or III GC who were treated in Seoul National University Bundang Hospital (Seongnam-si, Republic of Korea) from 2006 to 2013 were screened for inclusion. Among them, the tumor tissue samples of 14 patients were found inadequate for immunohistochemistry, thus excluded (Supplementary Figure 5). All 392 patients who were included in final analysis underwent curative surgical resection (R0 resection) with D2 lymph node dissection followed by fluoropyrimidine (FP)-based adjuvant chemotherapy (5-fluorouracil (5-FU), capecitabine, or S-1 with cisplatin, if clinically indicated). Clinicopathologic characteristics, including overall survival (OS) and disease free survival (DFS) were obtained retrospectively from medical records and pathology reports. OS was defined as the time from surgery to the date of death by any cause or censoring, and DFS was defined as the time from surgery to the date of recurrence of disease.

Surgically resected GC specimens from patients were formalin-fixed and paraffin-embedded (FFPE). In all cases, one representative 2-mm core was selected from the invasive margin of the tumor by 2 experienced gastrointestinal pathologists (S.Y. and H.S.L.), and tissue microarrays (TMA) were constructed as described previously (Superbiochips Laboratories, Seoul, Republic of Korea) [36].

All human FFPE tissue samples were obtained from the archive of the Department of Pathology, Seoul National University Bundang Hospital. This study was approved by the institutional review board (IRB) of Seoul National University Bundang Hospital (IRB number: B-1606/349-308). Written patient consent and the consent process were waived by the IRB.

### IHC and EBV *in situ* hybridization (ISH)

IHC for CD8, Foxp3, E-cadherin, p53, and PD-L1 were performed with an automatic immunostainer (BenchMark XT; Ventana Medical Systems, Tucson, AZ, USA), according to the manufacturer's instructions. The IHC antibodies used in this study were as follows: CD8 (C8/114B, mouse monoclonal; Dako, Carpinteria, CA, USA); Foxp3 (236A/E7, mouse monoclonal; Abcam, Cambridge, UK); E-cadherin (clone 36, mouse monoclonal; BD Biosciences, Franklin Lakes, NJ, USA); p53 (DO7, mouse monoclonal; Dako); and PD-L1 (E1L3N, rabbit monoclonal; Cell Signaling Technology, Danvers, MA, USA). EBV ISH was performed with the INFORM EBV-encoded RNA (EBER) probe (Ventana Medical Systems).

To interpret the CD8 and Foxp3 staining, immunostained TMA slides were scanned, and the CD8^+^ and Foxp3^+^ cell densities (positive cell counts per mm^2^) in each core of TMA were counted by an Aperio image analysis system (Leica Biosystems, New Castle, UK). The CD8^High^ and CD8^Low^ groups were defined using the 25th percentile as the cut-off value, and median value was used as the cut-off for Foxp3.

All other immunostaining was interpreted by 2 pathologists (J.K. and H.S.L.) who were blinded to patient characteristics at the time of interpretation. Membrane staining of PD-L1 on more than 5% of tumor cells was interpreted as positive [20, 25]. For E-cadherin, complete loss of membrane staining or aberrant cytoplasmic staining was regarded as altered expression, while complete membrane staining as strong as that in the non-neoplastic epithelium was considered normal expression [37]. For p53, strong nuclear staining in more than 10% of tumor cells was interpreted as p53 overexpression/positive, and cases with less than 10% positive cells including those showing scattered positive or patchy positive cells were considered negative. [38].

### PD-L1 mRNA ISH

To detect *PD-L1* mRNA on the tissue microarray, the *PD-L1* RNAscope 2-plex detection kit (Advanced Cell Diagnostics, Hayward, CA, USA) was used according to the manufacturer's guidelines. The results were interpreted by 2 pathologists (J.K. and H.S.L.) according to the instructions in the RNAscope FFPE Assay Kit and were scored as described previously [39]: 0, no staining; 1, staining in < 10% of tumor cells, difficult to identify at 40×; 2, staining in ≥ 10% of tumor cells, difficult to identify at 20× but easy at 40×; 3, staining in ≥ 10% of tumor cells, difficult to identify at 10× but easy at 20×; 4, staining in ≥ 10% of tumor cells, easy to identify at 10×. A score of 4 was considered *PD-L1* overtranscription.

### Microsatellite instability (MSI)

MSI status was assessed by comparing the allele profiles of 5 markers (BAT-26, BAT-25, D5S346, D17S250, and S2S123) in tumor cells to those in matched normal samples. The polymerase chain reaction (PCR) products from the FFPE samples were analysed with a DNA autosequencer (ABI 3731 Genetic Analyzer; Applied Biosystems, Foster City, CA, USA) according to a previously described protocol [40].

### Processing and analysis of genomic data

We used the publicly available level 3 data from TCGA downloaded from the UCSC Cancer Browser (http://genome-cancer.ucsc.edu) on June 3, 2015, which included clinical information and mRNA expression data obtained by RNAseq (Illumina HiSeq V2 platform) of TCGA samples. The mRNA expression data were presented as reads per kilobase per million (RPKM) and were transformed into log 2 values for the analysis. MSI status was available for 414 stomach adenocarcinoma (STAD) samples, and EBV status was referenced from TCGA clinical data.

In addition, we obtained clinical and mRNA expression data from a SMC cohort (Samsung Medical Center, Seoul, Republic of Korea) shared by Cristescu and colleagues [17] (Gene Expression Omnibus, GSE62254) on April 17, 2015. The mRNA expression data were processed by the Affymetrix Human Genome U133plus 2.0 Array (Santa Clara, CA, USA).

For application of the TMIT classification to the genomic data, after merging the log 2-transformed RPKM values of *PD-L1* and *CD8A*, we divided TCGA and SMC cohort samples into 4 groups using the aforementioned cut-off values (the median for *PD-L1* and lower 25th percentile for *CD8A*).

### Statistical analysis

The associations between clinicopathological characteristics and TMITs were analysed by Chi-square, linear-by-linear, Kruskal-Wallis, and Wilcoxson/Mann-Whitney tests, if appropriate. Spearman rank correlation was used for the correlation analysis between PD-L1 IHC and *PD-L1* mRNA ISH. Kaplan-Meier analysis of OS and DFS according to TMIT and molecular classification was performed, and the significance of survival differences was assessed by the log-rank test. A *P-value* less than 0.05 was considered statistically significant. All statistical analyses were performed using SPSS statistics 22.0 (IBM, Armonk, NY, USA), with the exception of the genomic analysis and data presentation which were performed using the R statistical package 3.1.3 (http://www.r-project.org).

## SUPPLEMENTARY MATERIALS FIGURES AND TABLE


