# Supplementary material for: Clinicopathologic implications of immune classification by PD-L1 expression and CD8-positive tumor-infiltrating lymphocytes in stage II and III gastric cancer patients
Source: Oncotarget. 2017 Feb 17;8(16):26356–67. doi: 10.18632/oncotarget.15465 (PMC5432263; doi:10.18632/oncotarget.15465)
Supplement: Supplementary file 1 [file oncotarget-08-26356-s001.pdf]

Clinicopathologic implications of immune classification by PD-L1 expression and CD8-positive tumor-infiltrating lymphocytes in stage II and III gastric cancer patients

Supplementary Materials

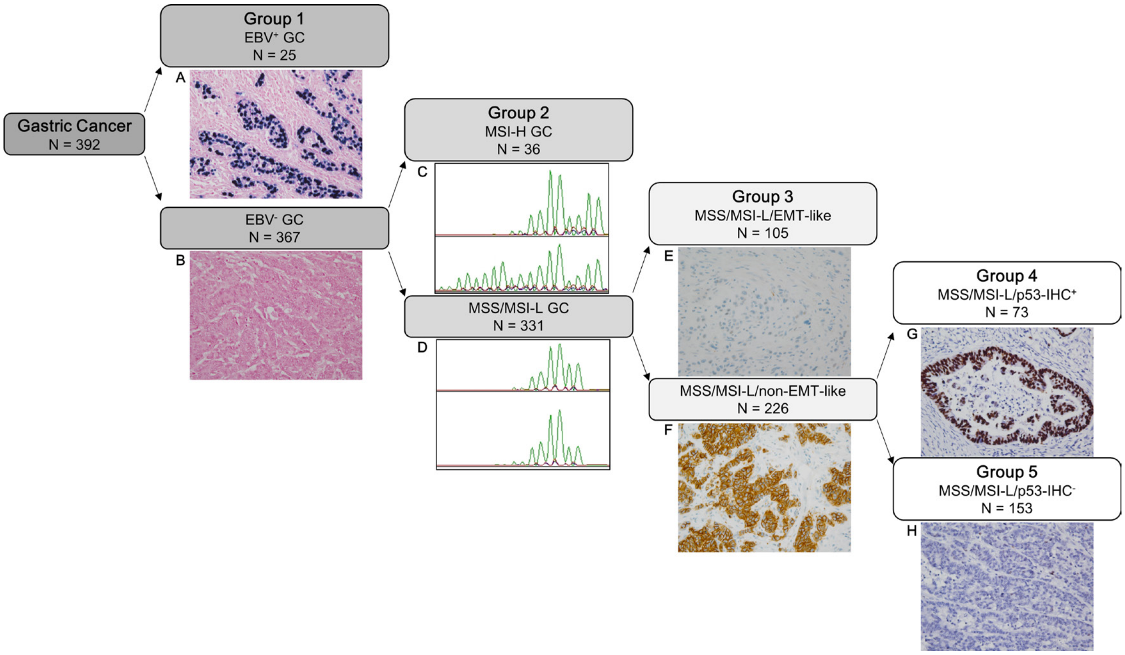

Supplementary Figure 1: Adaptation of molecular classification of gastric cancer (GC). After sorting out the Epstein-Barr virus (EBV)$^{+}$ GCs (group 1; A; EBV ISH), we sorted EBV^{-} GCs (B; EBV ISH) by microsatellite instability (MSI) status. MSI-high (MSI-H) cases were categorized as group 2 (C), and microsatellite stable (MSS)/MSI-low (MSI-L) cases (D) were further classified as MSS/MSI-L/EMT-like cases (group 3; E; E-cadherin immunohistochemistry [IHC]) or MSS/MSI-L/non-EMT-like cases (F; E-cadherin IHC). Finally, the MSS/MSI-L/non-EMT-like cases were subclassified according to p53 IHC results as MSS/MSI-L/p53-IHC^{+} (group 4; G; p53 IHC) or MSS/MSI-L/p53-IHC^{-} (group 5; H; p53 IHC).

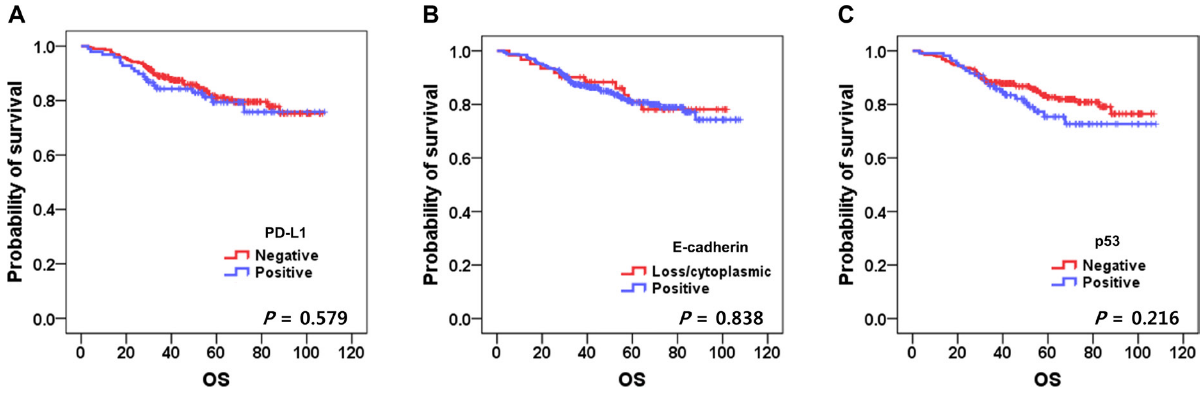

Supplementary Figure 2: Kaplan-Meier survival curves according to PD-L1, E-cadherin, and p53 immunohistochemistry (IHC) results. Survival analyses according to IHC of PD-L1 (**A**), E-cadherin (**B**), and p53 (**C**) showed no significant OS differences.

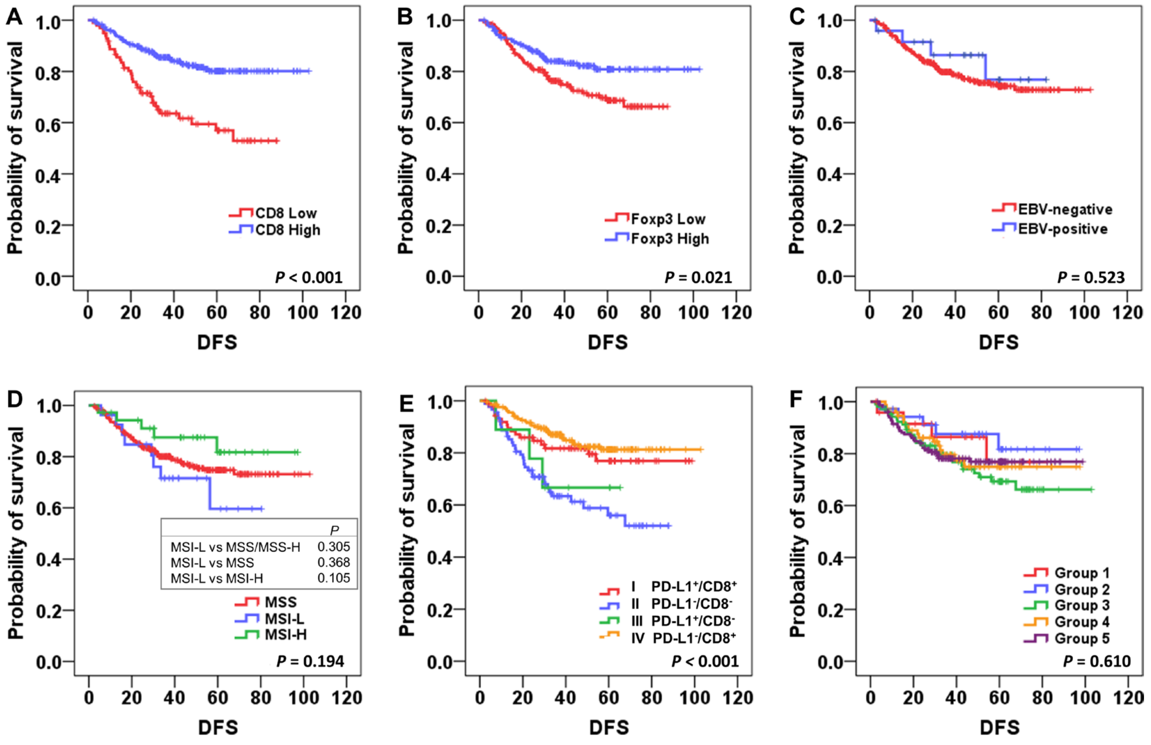

Supplementary Figure 3: Kaplan-Meier survival analysis of disease free survival according to major clinicopathologic features. CD8^{High} and Foxp3^{High} status were associated with better disease free survival (DFS) (**A** and **B**) ($P < 0.001$ and $P = 0.021$, respectively), and Epstein-Barr virus (EBV) status and MSI status were not significant prognostic factors (**C** and **D**). There were significant survival differences among the 4 tumour microenvironment immune types (TMITs; (**E**) $P < 0.001$), in contrast, no notable DFS differences were seen according to molecular classification (**F**).

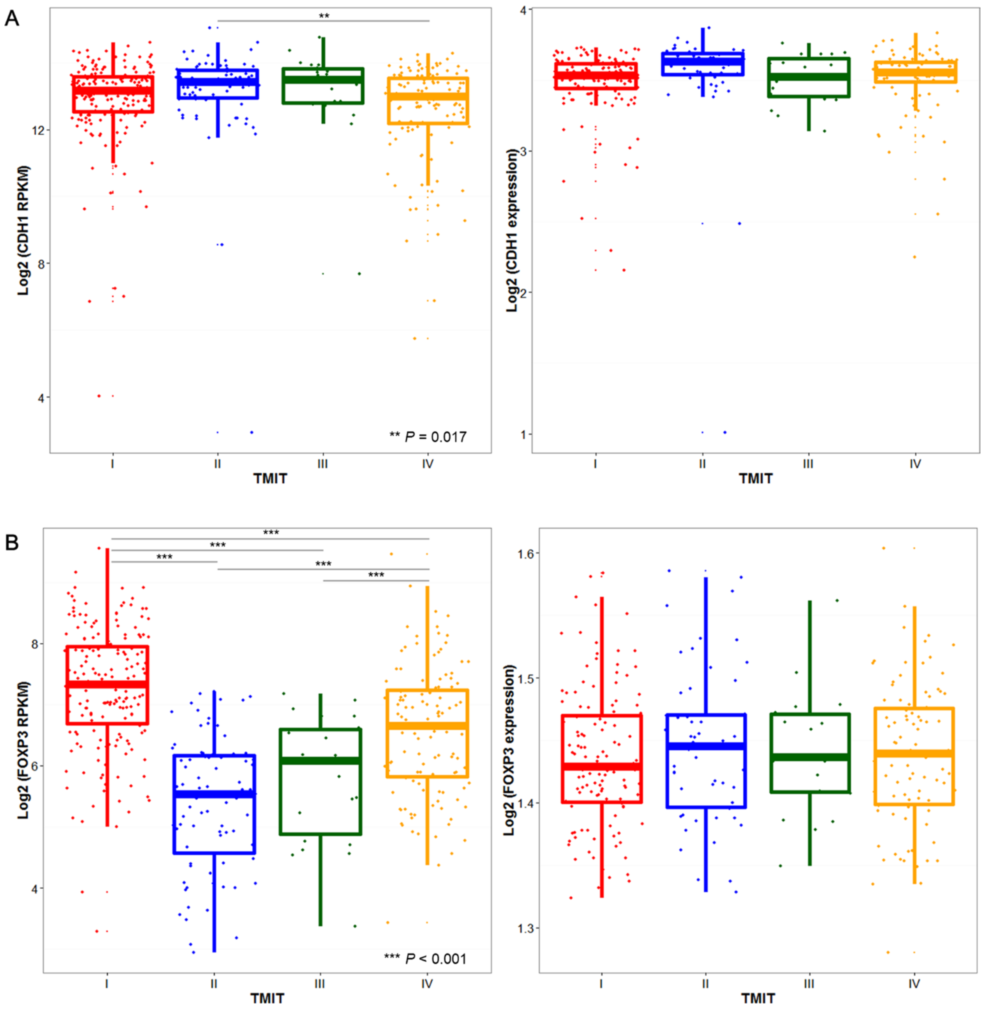

Supplementary Figure 4: Gene expression analysis of *CDH1* and *Foxp3* according to tumour microenvironment immune type (TMIT). The *CDH1* expression levels in each TMIT are shown (**A**; TCGA (left) and SMC (right)), and in TCGA dataset, TMIT IV showed lower *CDH1* expression than type II. There was no significant difference in *Foxp3* expression according to TMIT in the SMC cohort (**B**, right), whereas TMITs I and IV (**B**, left) showed higher Foxp3 expression than the other types in TCGA dataset.

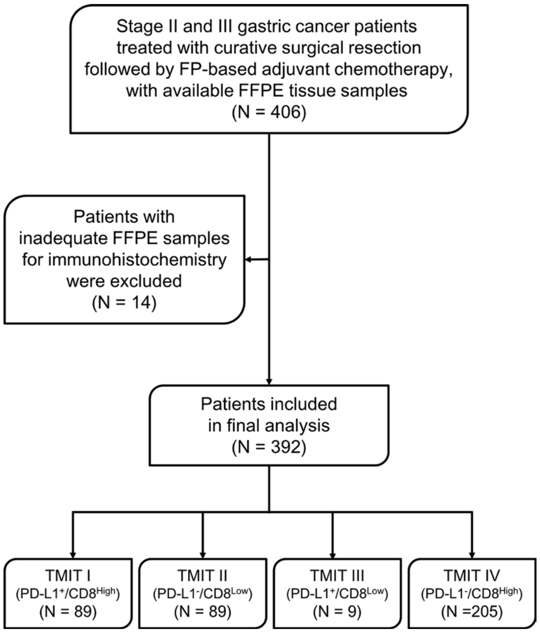

Supplementary Figure 5: Flow of study population throughout the study. Among the 406 stage II and III gastric cancer patients treated by surgical resection followed by FP-based adjuvant chemotherapy, 14 patients were excluded due to inadequate FFPE tissue samples for IHC study. As a result, a total of 392 patients were analysed.

Supplementary Table 1: Comparison between two methods of PD-L1 assessment

		PD-L1 IHC			Correlation coefficient
		Negative	Positive	Total	
PD-L1 mRNA ISH	0	280 (94.9%)	57 (58.2%)	337 (85.8%)	0.467
	1+	12 (4.1%)	15 (15.3%)	27 (6.9%)	
	2+	2 (0.7%)	7 (7.1%)	9 (2.3%)	
	3+	1 (0.3%)	5 (5.1%)	6 (1.5%)	
	4+	0 (0.0%)	14 (14.3%)	14 (3.6%)	
	Total	294 (75.0%)	98 (25.0%)	392 (100.0%)	

Abbreviation: IHC, immunohistochemistry; ISH, in situ hybridization.
